# Supplementary material for: Hyperoxidation of Peroxiredoxins and Effects on Physiology of Drosophila
Source: Antioxidants (Basel). 2021 Apr 15;10(4):606. doi: 10.3390/antiox10040606 (PMC8071185; doi:10.3390/antiox10040606)
Supplement: Supplementary file 1 [file antioxidants-10-00606-s001.zip › antioxidants-1150218-supplementary.pdf]

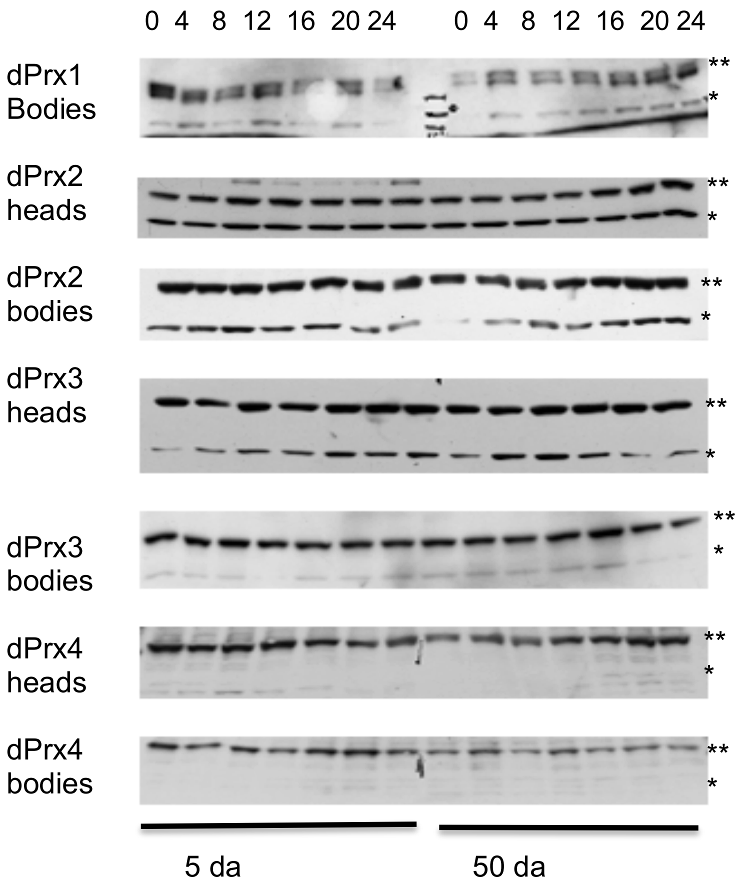

Figure S1. Immunoblot analysis of the daily changes in the peroxiredoxin dimer to monomer ratio in young (5 da) and old (50 da) flies. Samples were prepared from the heads and bodies of Canton male flies maintained under light/dark (LD) conditions and collected for analysis at 4 h intervals. Extracts were made using a non-reducing lysis buffer, as specified in Materials and Methods. Immunoblots were developed with antibodies specific for *Drosophila* peroxiredoxins 1-4. Shown are representative images. Positions of monomers (M) are marked by * and dimers (D) are indicated by **. 0-24 h is Zeitgeber time (ZT). where ZT0 is time of lights “on” and ZT12 is time of lights “off”.

A

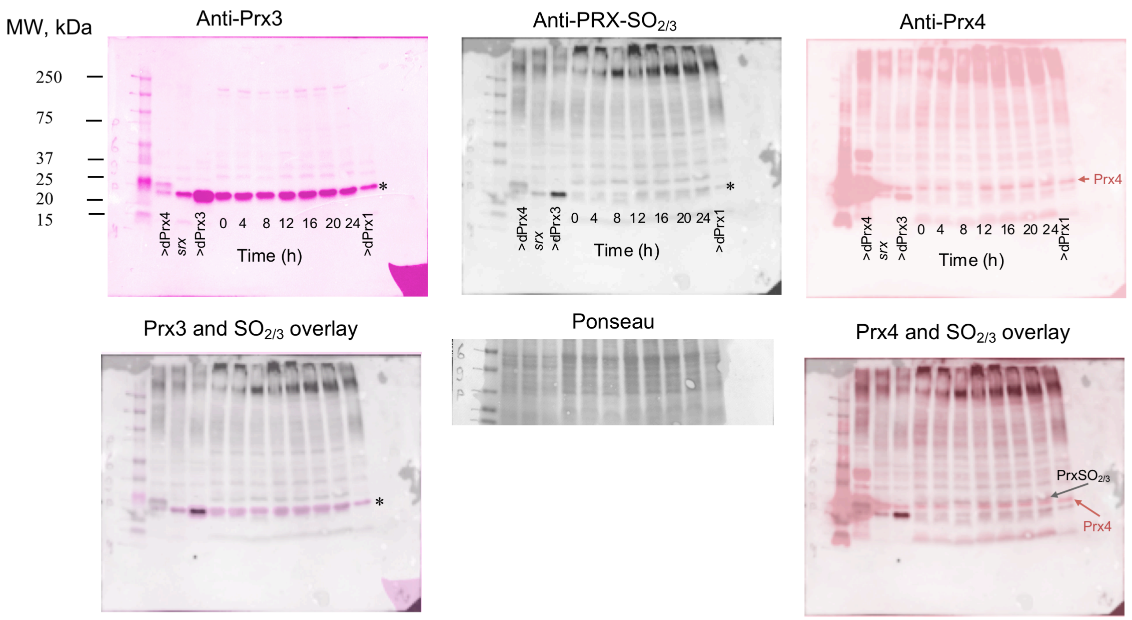
MW, kDa
Anti-Prx3
250
75
37
25
20
15
>dPrx4 srx >dPrx3 0 4 8 12 16 20 24 >dPrx1
Time (h)
Anti-PRX-$SO_{2/3}$
Anti-Prx4
Prx4
Prx3 and $SO_{2/3}$ overlay
Ponseau
Prx4 and $SO_{2/3}$ overlay
Prx$SO_{2/3}$
Prx4

B

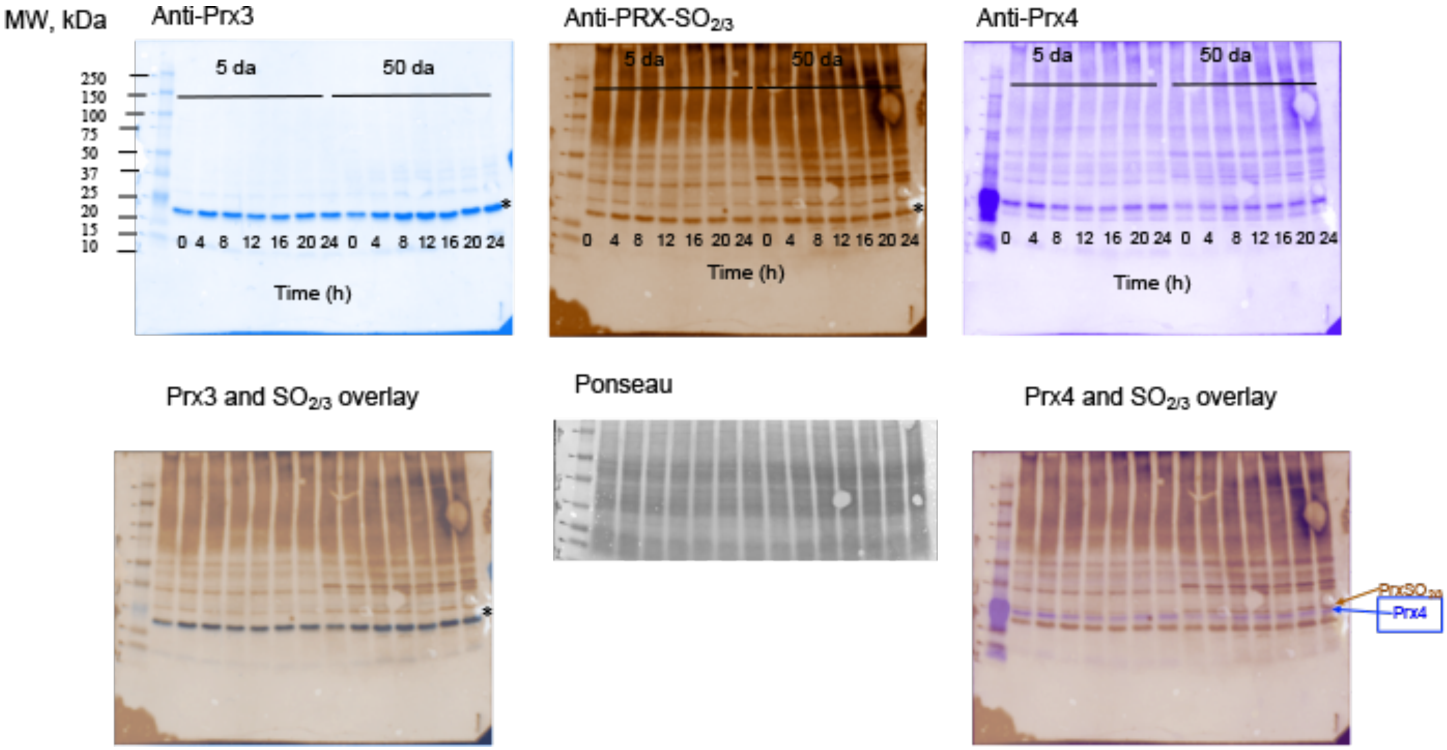
MW, kDa
Anti-Prx3
250
150
100
75
50
37
25
20
15
10
5 da
50 da
0 4 8 12 16 20 24 0 4 8 12 16 20 24
Time (h)
Anti-PRX-$SO_{2/3}$
Anti-Prx4
Prx3 and $SO_{2/3}$ overlay
Ponseau
Prx4 and $SO_{2/3}$ overlay
Prx4

C

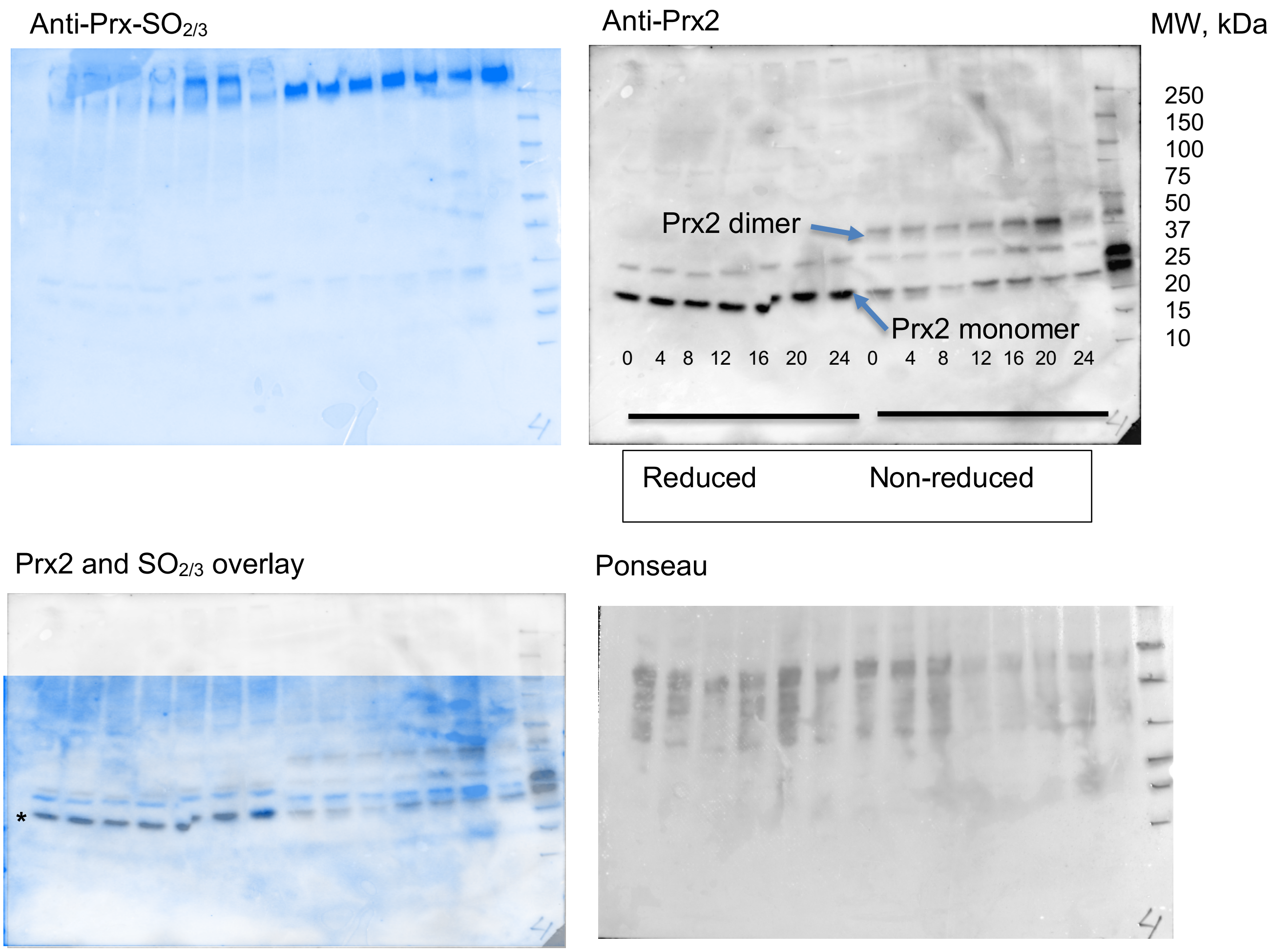

Figure S2. Analysis of the daily changes in peroxiredoxin hyperoxidation. Samples were prepared from the heads of 5 da old flies raised under LD conditions and collected at 4 hours intervals (0-24h) (**A** and **C**). Samples were prepared from bodies of 5 da and 50 da old flies (**B**). To control for antibody specificity, samples were also prepared from the whole flies overexpressing dPrx 4(>dPrx4), dPrx3 (>dPrx3), dPrx1 (>dPrx1) and a mutant of *Drosophila* homologue of sulfiredoxin (srx). **A and B:** Material was lysed using a reducing lysis buffer (50mM Tris pH 8.0, 150mM NaCl, 0.1%SDS, and 0.5% NaDeoxycholate) containing protease inhibitor cocktail (Roche) and 5% beta-mercaptoethanol. **C:** Protein lysates were prepared using non-reducing lysis buffer and divided into two equal portions. For gel electrophoresis, material of the first batch was prepared using non-reducing loading buffer, and the second - using loading buffer containing 5% beta-mercaptoethanol (reducing conditions).

Protein extracts were separated using 4-20% gradient gels (Bio-Rad). The protein ladder used was Precision Plus Protein™ Kaleidoscope™ Standards (Biorad). **A** and **B**: Immunoblots were developed with the anti-Prx-$SO_{2/3}$ antibody, reprobed with an antibody specific for *Drosophila* peroxiredoxin 4 (Anti-dPrx4), then stripped and reprobed with an antibody specific for *Drosophila* peroxiredoxin 3 (Anti-dPrx3). **C:** Immunoblots were developed with the anti-Prx- $SO_{2/3}$ antibody, then stripped and developed with anti-dPrx2 antibody. Images were obtained using ChemiDocTM Touch Imaging system (Bio-Rad) and analyzed using ImageLab software v.5.2.1 (Bio-Rad). The bottom panels illustrate overlaid images obtained with the anti-Prx-$SO_{2/3}$ and dPrx3 and dPrx4-specific antibodies. Arrows note positions of the dPrx-4 monomer (~25 kDa) and ~26 kDa band recognized by the anti-Prx-$SO_{2/3}$ antibody that show subtle difference in their migration. Asterisks indicate positions of overlapping signals obtained with both anti-dPrx3 and anti-Prx-$SO_{2/3}$ antibodies.

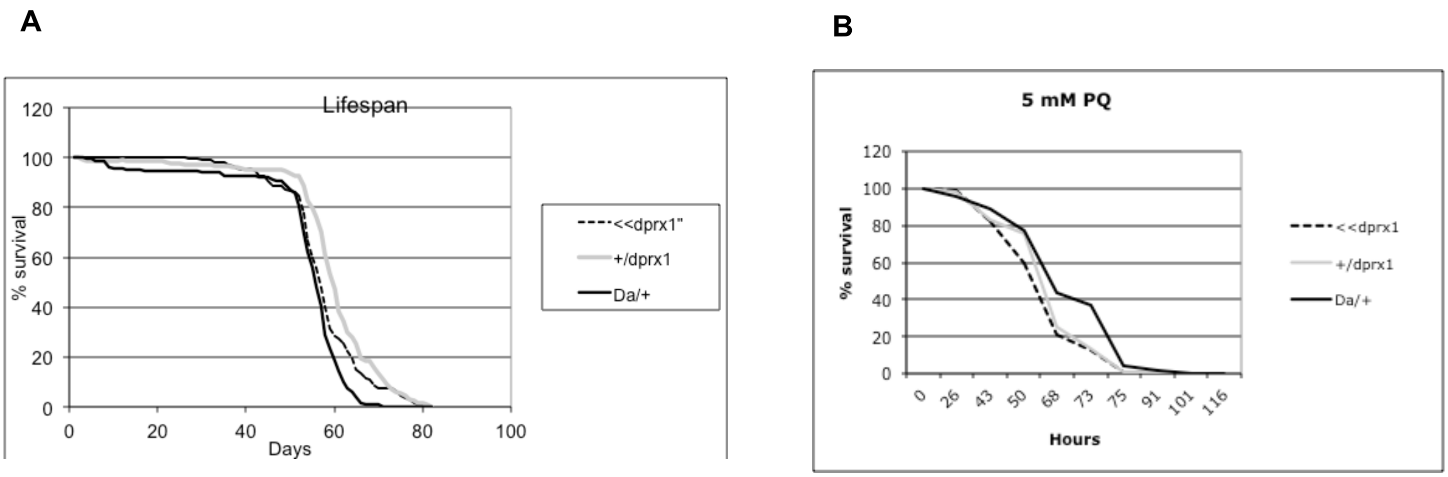

Figure S3. Survivorship of flies under-expressing dPrx1 under normal (A) oxidative stress conditions (B). Underexpression of dPrx1 was achieved by RNAi as described and demonstrated in Fig. 2. The levels of dPrx1 were not detectable by the immunoblot analysis. The results presented on the graphs are representative of 3 biological repeats for each treatment. Approximately 100 flies for each fly line were used in the experiments. Oxidative stress has been elicited by feeding flies 1% sucrose solution containing 5 mM paraquat. The survivorship has been analyzed by the log-rank test. No statistical significance has been determined between flies under-expressing dPrx1 (<<*dprx1*) and driver and transgene controls (+/*dprx1* and Da/+).

```
Human           ----------------------MGLRAGGTLGRAGAGRGAPEGPGPSGGAQGGSIHSGRI  38
Drosophila      MEFISHFLRATSRRTAALGPILQRNRSEIIQKQSLTNRQAFRRYRSSCSTMDTTVHSAGI
                                         *:     :: :.* * .   .* .: . ::**. *
Human           AAVHNVPLSVLIRPLPSVLDPAKVQSLVDTIREDP--DSVPPIDVLWIKGAQGGDYFYSF
Drosophila      DETHLVPMSVIQRPIPSVLDEQKVQSLMETIKNETSEDEVPPIDLLWISGSEGGDYYFSF
                  .* **:**: **:*****  *****::**:::.  *.*****:***.*::****::**
Human           GGCHRYAAYQQLQRETIPAKLVQSTLSDLRVYLGASTPDLQ-
Drosophila      GGCHRFEAYKRLQRPTIKAKLVKSTLGDLYHYMGSSAPKYLA
                *****: **::*** ** ****:***.**  *:*:*:*.
```

Figure S4. Alignment of amino acid sequences of human and *Drosophila* homologues of sulfiredoxin. Conserved cysteine residue in the active site of Srx protein responsible for reacting with peroxidatic cysteine residue of peroxiredoxin is shown by bold red.

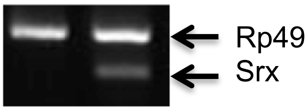

Figure S5. Analysis of CG6762 (Srx) expression in the *srx* mutant. RNA samples were isolated from control (yw) and the putative *srx* mutant (*srx*) followed by RT-PCR analysis with two different sets of primers that amplify the house-keeping rp49 gene to control for a loading and efficacy of RT-PCR reaction, as well as the CG6762 (Srx) gene. Primers for amplification the Srx mRNA were 5'-GGCATCGATGAGACCCACCT (forward) and 5'- TGAAGTAGTAGTCGCCTCCCT (reverse). Primers for rp49 gene are described in our publications [1]. No band corresponding amplification of the Srx fragment was detected in the material isolated from the *srx* mutant.

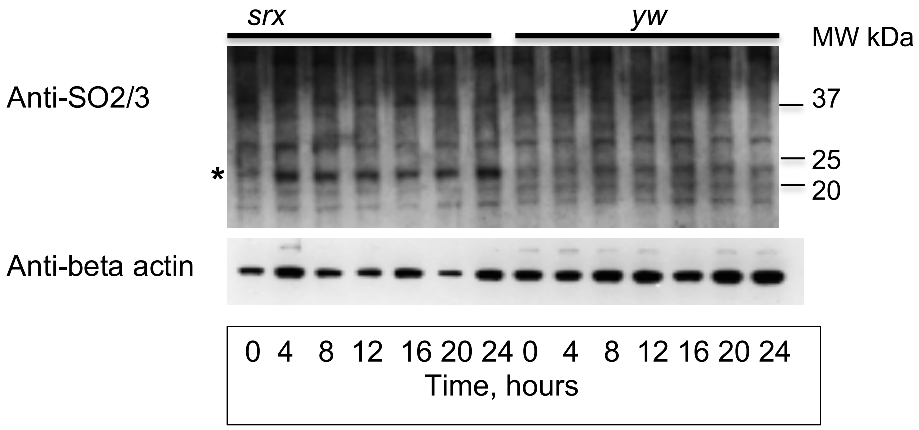

Figure S6. Analysis of the daily changes in peroxiredoxin hyperoxidation in the *srx* mutant. Samples were prepared from 5 da old flies raised under LD conditions and collected at 4 hours intervals (0-24h). Positions corresponding the monomers of over-oxidized Prx are marked by an asterisk. Analysis has been conducted as described in Fig. S2.

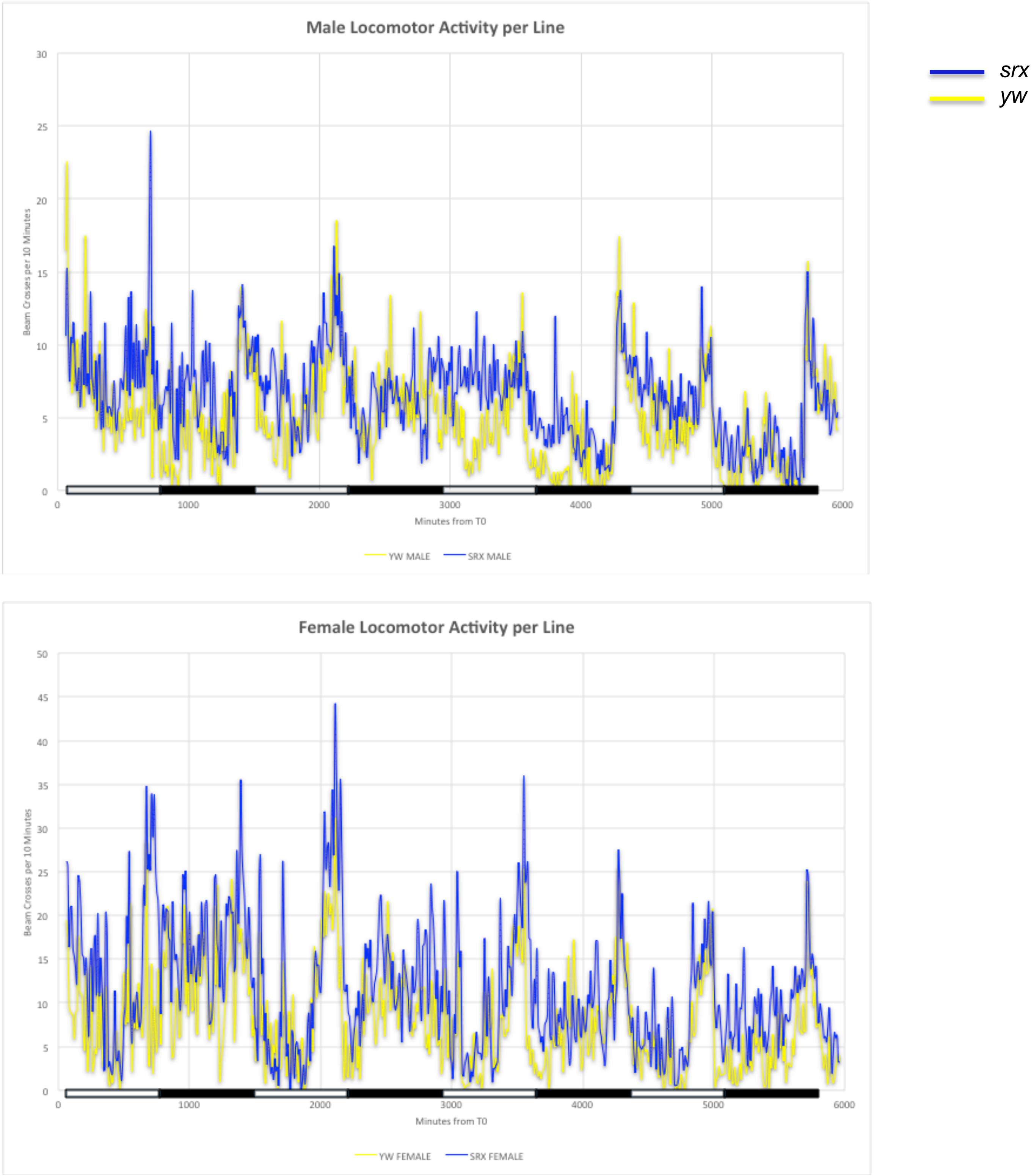

Figure S7. Analysis of the locomotor activity in the *srx* mutant. Shown is a representative data of activity of young (5 da) flies. Alternating light and dark boxes indicate the 12 hr light/ dark cycles. Eight tubes with flies were used for each fly line. Beam crosses were recorded at 10 min intervals.

[1] K. Michalak, W.C. Orr, S.N. Radyuk, Drosophila peroxiredoxin 5 is the second gene in a dicistronic operon, Biochem Biophys Res Commun, 368 (2008) 273-278.
